# Dr. W.H. Dwinelle

**Published:** 1896-03

**Authors:** 


### DR. W. H. DWINELLE.

Died in Cazenovia, N. Y., February 13, 1896, Dr. W. H. Dwinelle, in the 75th year of his age. For the past four or five years Dr. Dwinelle's health had been failing. He was all his lifetime a very hard worker, both physically and mentally, and doubtless to this strain is due in a large measure the break-down of his nervous system, which he with a great effort resisted for two or three years. His system finally gave away, however, and for the past three years he had been unfit for any work, either physical or mental.

Dr. Dwinelle has been a prominent worker in the profession for over fifty years, and for thirty years or more was a man of great influence. He did much in behalf of the profession in its early days, and was a great force during the time of the transition in the profession by which it passed from a mere trade to a profession.

His aspirations were always for the highest professional attainments, and he labored to that end, not only for himself, but his efforts were always manifested by his practice, his pen and in discussion for the elevation of his fellow-practitioners to the highest point of excellence. He was cotemporary and a co-worker with that band of noble men to whom dentistry owes so much, mainly Dr. Chapin A. Harris, Robert Arthur, Elisha Townsend, Solyman Brown, Eleazer Parmley, Elisha Baker, Joshua Tucker, Edward Harwood, J. F. Flagg, J. D. White, and a large number of others, all of whom did grand pioneer work in the days of seed-sowing.

Dr. Dwinelle was a man of large mental caliber, of commanding presence, affable and genial in his intercourse with professional brethren, and always ready to communicate whatever he had that would be of value to others. He had a thorough knowledge of general medicine, and always made that subservient to his chosen department. He served his day and generation well. But few in the profession of to-day knew Dr. Dwinelle personally, but the memory of what he did should everywhere be kept fresh in mind, and the example he set in professional work should he observed by all.

